# How to improve the outcomes of elderly acute myeloid leukemia patients through allogeneic hematopoietic stem cell transplantation

**DOI:** 10.3389/fimmu.2023.1102966

**Published:** 2023-05-03

**Authors:** Shan Jiang, Han Yan, Xuan Lu, Ruowen Wei, Haoran Chen, Ao Zhang, Wei Shi, Linghui Xia

**Affiliations:** Department of Hematology, Union Hospital, Tongji Medical College, Huazhong University of Science and Technology, Wuhan, China

**Keywords:** acute myeloid leukaemia (AML), elderly patients, allogeneic hematopoietic cell transplantation, conditioning regimen, GvHD, relapse

## Abstract

In recent years, with the gradual advancement of haploidentical transplantation technology, the availability of donors has increased significantly, along with the widespread use of reduced-intensity conditioning and the improvement of nursing techniques, giving more elderly acute myeloid leukemia (AML) patients the chance to receive allogeneic hematopoietic stem cell transplantation. We have summarized the classic and recently proposed pre-transplant assessment methods and assessed the various sources of donors, conditioning regimens, and post-transplant complication management based on the outcomes of large-scale clinical studies for elderly AML patients.

## 1 Introduction

The incidence of acute myeloid leukemia (AML) rises sharply after the age of 50 (1). According to the National Cancer Institute, the median age at diagnosis of AML patients is 68 years old (2). Compared with younger AML patients, elderly AML patients are in a poorer physical condition, have more comorbidities, and have a higher proportion of adverse prognostic factors such as unfavorable cytogenetics or secondary acute myeloid leukemia (3, 4).

Most elderly patients with AML are not suitable for intensive chemotherapy, and those who only receive best supportive care have a poor prognosis, with overall survival (OS) rates of 15% at 1 year and 2% at 5 years. In recent years, the use of venetoclax plus hypomethylating agents (HMAs) such as azacitidine or decitabine in elderly patients with AML who are not suitable for intensive chemotherapy has been shown to have a high remission rate [67% complete remission (CR)/incomplete blood count recovery (CRi)] and is well tolerated, with a long-term overall survival (OS) benefit (median OS of 17.5 months with a median follow-up of 15.1 months) (5–8). In the past, due to the high non-relapse mortality (NRM) and treatment-related mortality (TRM), most physicians restricted allo-HSCT to individuals under 60 years old (9). However, with the advancement of haploid hematopoietic stem cell transplantation (haplo-HSCT) technology and the development of post-transplantation complication management, more and more elderly AML patients are now able to undergo allo-HSCT. According to the Center for International Blood and Marrow Transplant Research, from 2016 to 2020, 66% of patients who received allo-HSCT were over the age of 50, and 27% were over the age of 65. The proportion of patients over the age of 65 who received allo-HSCT increased from 4% in 2005 to 27% in 2020 (10).

This article reviews the process and the management of post-transplant complications in elderly AML patients, including various assessment methods before allo-HSCT, donor sources, graft-*versus*-host disease (GVHD), and management of post-HSCT recurrence, other complications control, *etc.*

## 2 Pre-transplant assessment

### 2.1 Patient-related factors

A large-scale survival analysis by Joseph Maakaro et al. regarding the patient’s age upon transplantation came to the conclusion that age itself is not a contraindication to allo-HSCT in AML patients (11, 12). However, patients over the age of 80 should not receive allo-HSCT (13).

Clinical assessment methods like the Hematopoietic Cell Transplant Composite Risk (HCT-CI), Eastern Cooperative Oncology Group (ECOG), Karnofsky Performance Scale, and European Group for Blood and Marrow Transplantation are frequently used when evaluating the patients’ current physical performance (14) (**Table 1**). The Comprehensive Geriatric Assessment (CGA) and the Instrumental Activities of Daily Living (IADL) scales, age, comorbidities (Charlson Comorbidity Index), and albumin (IACA) index may be able to fill this gap since there is still no widely accepted scoring system specifically for elderly transplant patients (**Table 1**).

**Table 1:** Methods of pre-transplant assessment.

Assessment method	Assessment content	Survival
HCT-CI (15)	Heart disease, liver disease, inflammatory gastrointestinal disease, metabolic disease, and other complications included in HCT-CI. Articles by Sorhol ML et al. (15) contain detailed scoring criteria	2-year OS 0 points: 71% 1 to 2 points: 60% ≥3 points: 34%
ECOG (16)	Grade 0: Fully active, able to carry on all pre-disease performance without restriction Grade 1: Restricted in physically strenuous activity but ambulatory and able to carry out work of a light or sedentary nature, *e*.*g*., light housework, office work Grade 2: Ambulatory and capable of all self-care but unable to carry out any work activities, up and about more than 50% of waking hours Grade 3: Capable of only limited self-care, confined to bed or chair more than 50% of waking hours Grade 4: Completely disabled, cannot carry on any self-care, totally confined to bed or chair Grade 5: Dead	Significantly detrimental impacts of ECOG (0/1 and ≥2) on OS (HR = 1.72, *P* = 0.001) (17)
Karnofsky Performance Scale (KPS) (18)	100: Normal, no complaints; no evidence of disease 90: Able to carry on normal activity; minor signs or symptoms of disease 80: Normal activity with effort, some signs or symptoms of disease 70: Cares for self but unable to carry on normal activity or to do active work 60: Requires occasional assistance but is able to care for most of personal needs 50: Requires considerable assistance and frequent medical care 40: Disabled; requires special care and assistance 30: Severely disabled; hospitalization is indicated although death is not imminent 20: Very ill; hospitalization and active supportive care necessary 10: Moribund 0: Dead	The KPS score is not an independent predictor of survival outcomes for elderly AML patients (19)
mEBMT (20)	0 points for mEBMT are patient’s age at allo-HSCT <20, CR1, MSD and except female donor/male recipient (FDMR) 1 point for mEBMT are patient’s age at allo-HSCT is 20–40, CR>1, except MSD and FDMR 2 points for mEBMT are patient’s age at allo-HSCT >40 and no CR The mEBMT value is obtained by adding up the above-mentioned scores	5-year OS 0/1 point: 69% (51%–86%) 2 points: 58% (43%–73%) 3 points: 45% (30%–60%) 4 points: 33% (20%–47%) 5/6 point: 26% (11%–41%)
CGA (21)	Comorbidity: CCI, KF Scale, HCT-CI, CIRS-G Functional status: Zubrod PS, FI, Katz ADL, Lawton IADL, SF-36 PCS Mental health: SF-26 MCS Nutritional status: BMI, albumin, mg/dl	Significantly improved sensitivity through the use of multiple methods allows for a more accurate prediction of outcomes following HSCT
IACA index (22)	0 points for IADL scale of 8, age ≤75, CCI score <3, serum albumin (g/dl) ≥3.4 1 point for IADL scale of 6 to 7, age >75, CCI score ≥3, serum albumin (g/dl) <3.4 2 points for IADL scale ≤5 The IACA value is obtained by adding up the above-mentioned scores Low risk: score 0 Intermediate risk: score 1 to 2 High risk: score ≥3	2-year OS Low risk: 47.7% Intermediate risk: 20.2% High risk: 0%
FV Michelis et al. (23)	Favorable-risk group: CR1 ≥6 months, age <55, and HCT-CI score 0–3 Intermediate-risk group: CR1 ≥6 months, age <55, and HCT-CI score 4–5 High-risk group: CR1 <6 months, independent of age, as well as CR1 ≥6 months and age ≥55 years	5-year OS Favorable risk group: 53% (95% CI = 35%–68%) Intermediate risk group: 31% (95% CI = 11%–54%) High-risk group: 6% (95% CI = 1%–22%)
Laure Veltri et al. (24)	Low-risk group: MRD-negative patients with ≤3 other adverse prognostic factors (and no MMC) Intermediate-risk group: MRD-negative patients with multiple adverse factors or patients with detectable leukemia (or MMC) with ≤1 adverse factors High-risk group: detectable leukemia and more than one additional adverse prognostic factors (or a MMC) Seven adverse prognostic factors: history of lack of hematological recovery during induction or consolidation chemotherapy prior to transplantation, high-risk genetics, donor–recipient human leukocyte antigen (HLA) matching <13/14, history of cardiovascular disease, conditioning regimen, detectable leukemia at the time of HSCT and MMC	2-year OS Low risk group: 76.2% (CI = 63.3–85.6) Intermediate risk group: 32.2% (CI = 22.1–44.3) High-risk group: 7.7% (CI = 3.1–17.8)
DRSS (25)	AML is graded according to *de novo* or secondary disease, disease status (CR1, CR2, refractory) and cytogenetics (favorable, intermediate, adverse, unknown). There is an online interface for calculating DRSS categories (https://joshuafein.shinyapps.io/drss_calculator/)	The unadjusted OS curve is available alongside the risk categories on the online site

CGA, comprehensive geriatric assessment; CCI, Charlson comorbidity index; KF Scale, Kaplan–Feinstein Scale; HCT-CI, hematopoietic cell transplantation comorbidity index; CIRS-G, cumulative illness rating scale—geriatrics; Zubrod PS, Zubrod performance status; FI, Fried frailty index; Katz ADL, Katz activities of daily living; Lawton IADL, Lawton instrumental activities of daily living; SF-36 PCS, physical component summary of the SF-36; SF-26 MCS, mental component summary of the SF-36; BMI, body mass index; dl, deciliter; IADL, instrumental activities of daily living; IACA, instrumental activities of daily living scales, age, comorbidities, albumin; OS, overall survival; CR1, first complete remission; CI, confidence interval; MRD, minimal residual disease; MMC, major medical complications; HLA, human leukocyte antigen; DRSS, disease risk stratification system; AML, acute myeloid leukemia.

For elderly AML patients with normal ECOG, CGA includes tests for functional status, frailty, disability, and mental health, which aid in identifying heterogeneity (21, 26–28). According to studies, the aberrant CGA evaluation percentage in patients with normal ECOG ranges from 23.7% to 40% (26). However, CGA is complicated as there are no set standards (22).

China’s Beijing Hospital suggests the IACA index as a straightforward and useful CGA tool. According to several research, the IACA index can accurately predict the prognosis of elderly patients with AML (22). However, the publication that advocated this assessment approach used retrospective research and included a smaller number of older patients, which may have impacted the accuracy of the results, particularly for the high-risk category.

There are not many studies accessible, and it is unknown whether they are valid. A clinical trial is currently being conducted to evaluate the relationship between CGA and general health, quality of life (QOL), and prognosis in older transplant patients (NCT04375579).

### 2.2 Disease-related factors

It is controversial whether elderly patients in first complete remission (CR1) or second complete remission (CR2) benefit from HSCT. Fotios V. Michelis and colleagues conducted a follow-up analysis of 196 patients over 60 who had their first HSCT in CR2 between 2001 and 2012. According to the results, this group’s 3-year OS following HSCT was 42%. Patients with unfavorable cytogenetics among them had a cumulative incidence of relapse (CIR) of up to 70% and a 3-year OS of only 25%. Thus, it was shown that individuals with an adverse cytogenetic risk have very little to gain from transplantation and that HSCT has the ability to cause long-term remission in middle-aged and elderly AML patients who are in CR2 (29). Studies by Paul M. Armistead et al. (30) also verified the finding that HSCT does not significantly exceed chemotherapy alone in treating elderly patients with CR2 (*p* = 0.43; median survival time, 5.2 months). In comparison with chemotherapy alone, a large-scale clinical study by Burnett et al. (31) revealed significant survival improvements for young AML patients (median age, 38) who were at CR2 and at medium to high cytogenetic risk. (The 5-year OS is, respectively, 16% and 42%.) This discrepancy between young and elderly patients could be explained by the elderly CR2 patients’ increased HCT-CI, many chemotherapy regimens, severe infections, and other comorbidities that led to a more serious organ damage and higher NRM (32). Almost all of the patients in CR2 had a hazard ratio (HR) for NRM that were more than two times higher than those who were older in CR, which led to a dismal survival outcome.

To divide the patients into several risk groups, FV Michelis et al. suggested calculating multiple scores for age, the duration of the patient’s first remission, and the HCT-CI score. Selecting individuals who might benefit from HSCT is made possible by the substantial differences in prognosis between groups (23) (**Table 1**).

After receiving more chemotherapy, more than 30% of elderly AML patients were still positive for minimum residual disease (MRD), and their MRD remained high before receiving HSCT (11, 33, 34). Based on pre-transplant MRD status and the number of adverse prognostic factors, Lauren Veltri’s team categorized the patients into three risk groups to distinguish between those who demonstrably benefited from HSCT and those who benefited less or even encountered counterproductive effects (24), thus selecting the right population for HSCT as a result (**Table 1**).

Roni Shouval et al. developed the Disease Risk Stratification System (DRSS) based on histological diagnostic and response status at the time of HSCT as well as molecular and cytogenetic data, and it is optimized to take into account population heterogeneity, improve power, and increase generalizability. The leading allogeneic transplant indication, AML, is divided by ontology (*de novo vs*. secondary), cytogenetics, and mutations for the first moment in DRSS, a global prognostic system. DRSS covers 15 hematological malignancies, including AML, and the histological and remission status are combined with each other to form 55 levels, culminating in five risk strata: low, intermediate-1, intermediate-2, high, and very high risk categories. The prognosis of patients is predicted by a rising trend in recurrence, which raises the incidence of mortality in each stratum (25) (**Table 1**).

## 3 Donor sources

The immune systems of older matched sibling donors (MSD) may have degraded, resulting in low levels of circulating naïve T cells needed for immune responses and less effective graft-*versus*-leukemia (GVL) (35, 36). Additionally, the HR for overall mortality rises by 5.5% for every decade that the donor age rises (37). It has been demonstrated that grafts from matched unrelated donors (MUD) and haplo-related donors (HRD) have comparable survival. The incidences of grades II–IV acute GVHD (aGVHD), moderate–severe chronic GVHD (cGVHD), and NRM were all decreased in elderly patients who received younger HRD derived from youngsters (37, 38). Multiple other research have found that HRD-HSCT improves the long-term disease-free survival (DFS) in older individuals by having a higher GVL effect that decreases recurrence. The recurrence rate for HRD-HSCT was 15.4%, for MSD-HSCT it was 28.2%, and for MUD it was 49.9% (39–41). It typically takes 4–6 weeks for a successful MUD (bone marrow bank and cord blood source) match (42). Peripheral blood (PB) grafts can be used as a high-risk group for relapse, but it is challenging for MUD-sourced providers to apply excess apheresis stem cells for donor lymphocyte infusion (DLI) or natural killer cell-based therapy to avoid recurrence (43). In conclusion, for older patients who want a transplant as soon as feasible or who have an older MUD, selecting a young, related haploidentical transplant is not a bad option.

## 4 Conditioning

### 4.1 Fludarabine + melphalan/fludarabine + busulfan reduced-intensity conditioning

The fludarabine + melphalan (FM) and fludarabine + busulfan (FB) regimens are the two most commonly used combinations for reduced-intensity conditioning (RIC) (44). In 404 AML patients over the age of 60, Stefan O. Ciurea et al. carried out a comparative analysis of outcomes after HSCT. Fludarabine + IV busulfan area under the concentration–time curve (AUC) 5,000/day for 4 days (Bu20000), fludarabine + IV busulfan AUC 4,000/day for 4 days (Bu16000), fludarabine + IV busulfan AUC 100 mg/m^2^ (FM100), and fludarabine + IV busulfan 140 mg/m^2^ (FM140) were administered as conditioning regimens to the enrolled patients, respectively. The analysis’ results showed that the FM100 group had a significantly better long-term survival without an increase in recurrence rate (RR) and wit a lower NRM, particularly in patients with poor performance status, those who were older than 65 years, and those who had no active disease before transplantation. The outcome indicators of the other three groups showed no significant difference. However, there were no significant differences in the outcome measures across the four conditioning groups for those who had a high-risk AML (45). In addition, compared with FB-RIC, patients who received FM-RIC had a greater likelihood of developing complete donor chimerism (CDC) at +30 days following transplantation, which was linked to superior post-transplant outcomes (24) (**Table 2**).

However, Zheng Zhou et al. performed an extensive retrospective study with a median age of 61 years. The results showed that the FM regimen had a higher early NRM than the FB regimen, but the FM regimen had better long-term OS and leukemia-free survival (LFS). For elderly AML patients with higher HCT-CI, the team thinks FB may be the better decision. FM might be a wiser choice for younger patients who have a higher risk of recurrence (46) (**Table 2**).

**Table 2:** Comparison of the survival outcomes between different conditionings.

Study	Type	Disease (n)	Median age (range years)	Conditioning	OS	NRM	Relapse	LFS
Stefan O Ciurea et al. (45) 2020	Retrospective	AML (89)	67 (60–79)	FM100	5-year 49%	3-year 19%	3-year 32%	5-year 49%
		AML (78)	64 (60–76)	FM140	5-year 30%	3-year 39%	3-year 32%	5-year 30%
		AML (131)	64 (60–73)	Bu≥20000	5-year 34%	3-year 35%	3-year 30%	5-year 34%
		AML (106)	65 (60–77)	Bu16000	5-year 23%	3-year 21%	3-year 55%	5-year 23%
Zheng Zhou et al. (46) 2020	Retrospective	AML (384)	59 (19–76)	FM (FM100 or FM140)	5-year 29%	3-year 32%	3-year 33%	5-year 28%
		AML (627)	61 (18–76)	FB (3.2 or 6.4 mg/kg)	5-year 27%	3-year 19%	3-year 52%	5-year 25%
Florent Malard et al. (47) 2017	Retrospective	AML (159)	52 (19–76)	FLAMSA TBI, 4 Gy	2-year 62%	2-year 19.4%	2-year 21.2%	2-year 58.8%
		AML (106)	61 (25–74)	FLAMSA-Bu (6.4 mg/kg)	2-year 46.7%	2-year 31.1%	2-year 25.7%	2-year 43.2%
Charles Craddock et al. (48) 2021	Prospective	AML (82) MDS (40)	59 (22–75)	FMA/FBA/FB-ATG	2-year 58.8%	2-year 21%	2-year 29.5%	2-year 48.7%
		AML (82) MDS (40)	59 (22–75)	FLAMSA-Bu	2-year 60.9%	2-year 20%	2-year 26.7%	2-year 54.2%

OS, overall survival; NRM, non-relapse mortality; LFS, leukemia-free survival; AML, acute myeloid leukemia; FM100, fludarabine + melphalan 100 mg/m^2^; FM140, fludarabine + melphalan 140 mg/m^2^; Bu ≥ 20000, fludarabine + IV busulfan AUC ≥ 5,000/day × 4 days; Bu16000, fludarabine + IV busulfan AUC 4,000/day × 4 days; FB, fludarabine + busulfan; FLAMSA TBI 4 Gy, fludarabine/amcridine/cytarabine–busulfan + total body irradiation 4 Gy; FLAMSA-Bu, fludarabine/amcridine/cytarabine–busulfan–busulfan; FMA, fludarabine/melphalan/alemtuzumab; FBA, fludarabine/busulphan/alemtuzumab; FB-ATG, fludarabine/busulphan/antithymocyte globulin; MDS, myelodysplastic syndromes.

### 4.2 Fludarabine/amcridine/cytarabine-busulfan or total body irradiation RIC

Recurrence is the most frustrating issue for RIC-HSCT patients. There is no discernible difference in patient outcomes between total body irradiation (TBI)-based and Bu-based regimens, and the fludarabine/amcridine/cytarabine-busulfan (FLAMSA) conditioning regimen can reduce the RR of patients with intermediate- and high-risk AML and effectively control the primary disease according to the study by Florent Malard et al. (47, 49, 50). Charles Craddock et al., however, had a different viewpoint. According to their team’s findings, there was no meaningful difference in 2-year OS, event-free survival (EFS), TRM, or CIR between patients receiving the FLAMSA-Bu regimen *versus* those receiving the RIC regimen based on fludarabine. As a consequence, the FLAMSA-Bu regimen, which was previously reported and frequently used, did not decrease the recurrence nor boost the prognosis in AML (48) (**Table 2**). A clinical trial comparing FLAMSA with clofarabine/Ara-C for EFS in patients with high-risk AML is currently being conducted (NCT01423175).

### 4.3 Other regimens of RIC

Additional ongoing clinical trials of RIC involve determining the safety and efficiency of a novel RIC regimen employing a low-dose Bu (9.6 mg/kg) plus Flu without anti-human thymus globulin (ATG) in elderly AML patients (NCT01828619). Additionally, some researchers suggested that using clofarabine instead of Flu with Bu and ATG as a new RIC regimen might be considered because it has a stronger anti-tumor effect than Flu without increasing the related toxicity, has better tolerance, and is more suitable for elderly patients. Clinical trials for this regimen are ongoing (NCT00863148).

### 4.4 Myeloablative conditioning and non-myeloablative conditioning

Since toxicity and TRM from myeloablative conditioning (MAC) regimens grow with age and ultimately result in worse outcomes, elderly patients frequently exhibit characteristics of frailty and have poor functional capacity. TBI 2 Gy plus Flu is the most popular regimen for non-myeloablative conditioning (NMC). According to reports, this regimen had a grade II–IV aGVHD incidence of 46% at 120 days and a 3-year cGVHD incidence of 72%, with GVHD being the chief reason, with a 3-year NRM of 7%. This regimen also had a 40% 3-year relapse/progression rate, a 28% 3-year relapse-related mortality rate, a 53% 3-year OS rate, and a 53% 3-year PFS rate (51).

## 5 GVHD prophylaxis

### 5.1 Calcineurin inhibitors

Several transplant centers consider GVHD prophylaxis with cyclosporine/tacrolimus, methotrexate, or mycophenolate mofetil (MMF) following transplantation to be a need; nonetheless, the effectiveness of calcineurin inhibitors (CNI) alone is limited, and 40%–60% of recipients still incur grades II–IV aGVHD (49, 50).

In an effort to increase the efficacy and lessen the toxicity, methods for combining several medicines with CNIs have been developed recently. For older patients receiving NMC-HSCT, sirolimus can be added to cyclosporine and MMF to prevent GVHD (52, 53). The rate of grades II–IV aGVHD at +100 days was 26%, while the 1-year NRM, OS, and PFS rates were 4%, 86%, and 77%, respectively.

### 5.2 Post-transplantation cyclophosphamide

In senior patients, the use of post-transplantation cyclophosphamide (PTCy) for the prevention of GVHD is well established and well tolerated (54, 55). The incidence of grades II–IV aGVHD and cGVHD in patients who received bone marrow (BM)-HSCT was 27% and 13%, respectively, within 200 days of HSCT (56). The grades II–IV aGVHD and cGVHD incidence in PB-HSCT was 33% and 13%, respectively (57).

After PB-HSCT, PTCy plus short-course sirolimus was more effective at preventing GVHD in elderly patients than PTCy alone. Grades II–IV aGVHD, grades III and IV aGVHD, and grades II–IV cGVHD had cumulative incidences (CI) of 46%, 15%, and 31%, respectively. The NRM is 4% within a year (58). Several studies examined the effectiveness of tacrolimus and MMF or uxolitinib and PTCy for the prevention of GVHD (NCT04669210).

PTCy +3 + 5 (CNI given on day 0, MMF given on day +1, and PT-CY given on days +3 and +5) is another regimen to prevent GVHD in elderly patients. A study’s findings showed a CI of 28% for patients above 60 years old with aGVHD grades II–IV, a CI of 3% for patients with aGVHD grades III and IV, an overall CI of 61% for cGVHD, and a CI of 18% for moderate to severe cGVHD (59).

### 5.3 ATG

ATG is the *in vivo* T cell-depleted (TCD) that is applied in clinical practice the most commonly. With 100-day CIs exceeding 50% for both cytomegalovirus (CMV) and Epstein–Barr virus (EBV) viremia, patients receiving ATG had a considerably increased risk of infection, raising the NRM. As a result, prior to HSCT, it is essential to evaluate the patient’s risk of recurrence (60). The effectiveness of RIC with PTCy along with ATG for the prevention of GVHD is now being evaluated in a randomized clinical trial (NCT02876679). For the purpose of preventing GVHD in patients receiving RIC-MUD-HSCT, some researchers have suggested combining PTCy with ATG on the basis of CNI; however, the efficacy of this combination has not yet been established (NCT03357159). For Haplo-PB-HSCT patients, some researchers have suggested a fresh regimen that combines low-dose ATG (5 mg/kg) and low-dose PTCy (one dose of PTCy, 50 mg/kg), which is anticipated to lower the risk of aGVHD and lower the incidence of viral reactivation (NCT03608059). Moreover, it has been considered that the effectiveness and safety of ATG and ATG-Fresenius for the prevention of GVHD be compared, and related research is now being done (NCT03631563).

### 5.4 *Ex vivo* TCD

The most typical clinical technique is to deploy the ClinicMACS system to select CD34 before performing *ex vivo* TCD on mobilized PBSC. This approach can considerably lower the incidence of severe GVHD without increasing RR. Furthermore, 14% of the occurrences of GVHD occur within 3 years (61); a clinical research is currently underway (NCT01189786).

GVHD incidence was decreased with PB-HSCT after human CD3+ T cell-depleted, but the recurrence was not enhanced (62). Pan-drawback TCDs of sluggish engraftment and immune reconstitution can be offset by using it in combination with ATG (63).

It is possible to minimize GVHD while maintaining the effects of GVL by depleting α/β T cells while retaining γ/δ T cells (64), although there are no data on its application in elderly patients. In patients receiving RIC-HSCT, a clinical trial is being conducted to assess the viability and safety of giving prophylactic CD45RAneg memory/effector T cells soon after transplantation (NCT05066412).

To investigate if they can prevent or diminish the incidence of GVHD, Treg from unrelated donors can also be altered in the lab until they eventually transform into fucosylated T cells (NCT02423915).

### 5.5 Other methods

A few new drugs that target novel targets, such as anti-CD154 monoclonal antibody, cytotoxic T-lymphocyte-associated antigen 4 globulin, and anti-CD25 monoclonal antibody, have significant research and application prospects in recent years. Moreover, human amniotic epithelial cells, ixazomib, itatinib, maraviroc, and bortezomib may have some effect on preventing GVHD (65) (NCT03082677, NCT03764228, and NCT04859946). Moreover, according to the authors, prebiotic galacto-oligosaccharide may help prevent GVHD by regulating the gut microbial activity, and pertinent clinical trials are currently being conducted (NCT04373057).

## 6 Prevention of relapse

### 6.1 Early discontinuation of immunosuppressive drugs

If CDC has not been reached at 4 weeks following HSCT and there is no severe aGVHD, start reducing immunosuppression for elderly AML patients, a high-risk group for relapse. Immunosuppression in older individuals who have reached CDC should be decreased 3 months after HSCT and stopped at 5 months (66). Therefore, physicians need to balance the production of severe GVHD with the induction of GVL consequences. According to some researchers, granulocyte colony stimulating factor reduces T cell reactivity, which may help distinguish between the GVL impact and GVHD (67). Unfortunately, no significantly prospective studies on a large scale have validated its efficacy.

### 6.2 Prophylactic DLI

DLI after +90 days helps lower the cumulative RR (5-year cumulative RR, 30.5%) and improves the outcomes (5-year OS, 69.8%) for high-risk AML patients. In DLI, the median CD3+ T celldosage was 3 × 10^6^/kg (compared with 1 × 10^7^/kg in MSD and 0.5 × 10^6^/kg in MUD) (68). The use of interferon α-2b in combination with prophylactic DLI has been indicated by several researchers to further diminish the RR and promote LFS, and related clinical trials are currently being conducted (NCT02568241). To assess the efficacy of siremadlin combined with proDLI for prophylaxis in high-risk patients, a phase Ib/II clinical trial is now being conducted (NCT05447663).

### 6.3 Targeted drug

In addition, the prevention of relapse includes the use of targeted medications. In elderly AML patients, HMA shows great efficacy and tolerance. The RR can be massively diminished by low-dose decitabine and recombinant granulocyte colony stimulating factor (15.0% *vs*. 38.3%) (69). AZA had a 1-year RFS of 46%, whereas CC-486’s oral formulation had a 1-year RFS of 54% and 72% for the 7- and 14-day dosing groups, respectively (70). Post-transplant maintenance therapy with sorafenib, midostatin, gitertinib, quizartinib, and crenolanib is an option for patients with FLT3-ITD/FLT3-TKD mutations (71). The most effective targeted agents for older individuals with FLT3 mutations will need to be identified in the future.

The Food and Drug Administration (FDA) approved ivosidenib and enasidenib as IDH1/2 mutations for the treatment of relapsed/refractory acute myeloid leukemia in 2018. Clinical research on its utilization as post-transplant maintenance therapy are ongoing, but no studies have yet established its involvement in the management of post-transplant relapse in elderly patients (NCT03515512 and NCT03728335). Histone deacetylase inhibitors, such as panobinostat, have showed excellent efficacy in preventing relapse in elderly patients who underwent HSCT (the CIR with panobinostat given prior to proDLI after HSCT was 35%, with 2-year OS and PFS of 50% and 49%, respectively (72)). Last but not the least, targeted drugs are typically utilized as therapy for 1 to 2 years following transplantation (73).

## 7 Management of post-transplant complications

### 7.1 Treatment of GVHD

#### 7.1.1 aGVHD

The conditioning regimens for elder AML patients are primarily RIC, in contrast to younger patients. The GVL effect, which reduces the likelihood of high recurrence in elderly patients, is its greatest advantage. The prevalence of grades III and IV GVHD can be greatly decreased with a PTCY-based GVHD prophylaxis. According to a recent retrospective research (74), the elderly patients’ survival outcomes benefit from the continuity of grade II aGVHD. Better DFS (HR: 0.36; *p* = 0.002) and OS (HR: 0.35; *p* = 0.003) for elderly patients with grade II aGVHD continue to show a substantial correlation, which may be attributable to controlled aGVHD and contributing to the GVL effect.

There is presently no standard second-line therapy for aGVHD, and systemic steroid therapy is the traditional first-line treatment (75, 76). As initial treatment for aGVHD, no combination medication has yet been proven to be better than corticosteroids alone. Alpha 1-antitrypsin plus corticosteroids are being evaluated in a recent phase III clinical trial for aGVHD (NCT04167514). As an alternative, coupling itolizumab or leflunomide with corticosteroids may also work well (NCT05263999 and NCT05443425).

Steroid-refractory acute graft-*versus*-host disease (SR-aGVHD), which involves about half of the patients, causes them to be resistant to steroids. The first medicine to be approved by the FDA for the treatment of SR-aGVHD was roxolitinib. According to the findings of the clinical trials REACH1 and REACH2, at least 50% of the SR-aGVHD patients (median ages 58 and 54 years, respectively) had an objective response (54.9%–62%). Other drugs for the treatment of SR-aGVHD in elderly patients, aside from roxolitinib, are currently undergoing clinical trials. T-Guard and ruxolitinib are being compared in a clinical trial for patients with grade III or IV SR-aGVHD (NCT04934670). In recently conducted clinical trials, the efficacy and safety of decidua stroma cells, itacitinib, and tocilizumab in the management of SR-aGVHD are being assessed (NCT04118556 and NCT04070781).

A study that focuses on extracorporeal photopheresis and mesenchymal stem cell infusion as a combination treatment for SR-aGVHD is also available (NCT05333029). Although it is still in the clinical research process, some researchers have suggested using umbilical cord mesenchymal stem cells alone or in conjunction with ruxolitinib to treat SR-aGVHD (NCT04738981 and NCT04744116).

We should concentrate on effective GVHD prophylaxis because the outcomes following grades III and IV aGVHD and SR-aGVHD are generally dismal for older patients.

#### 7.1.2 cGVHD

Based on prospective research (77), cGVHD is the primary factor in reducing relapse and improved DFS and OS following RIC-HSCT for elderly patients of bone marrow malignancies. A first-line therapy for cGVHD is systemic steroid combined with cyclosporin (78, 79). Ruxolitinib, ibrutinib, and belumosudil are the three drugs that the FDA has so far approved for use in the treatment of steroid-refractory chronic graft versus host disease (SR-cGVHD). According to a phase 3 open-label, randomized trial, ruxolitinib had a substantially higher ORR [49.7% *vs*. 25.6% (80)] at week 24 than the control group. Ibrutinib (PYC-1129) and belumosudil (ROCKSTAR) had ORRs of 67% and 76%, respectively. A phase 2, open-label, randomized, multicenter study is now recruiting participants to evaluate the effectiveness and tolerability of axatinib in SR-cGVHD (NCT04710576).

SHR0302 and prednisone are being examined in recent clinical research as the first-line therapy for moderate to severe cGVHD (NCT04146207). Ibrutinib and CD20 are also likely to succeed other treatments for cGVHD, and they are now being researched in phase II clinical trials (NCT04294641 and NCT04235036). In a recent study, BN101’s efficiency and safety for managing cGVHD are being examined (NCT04930562). A phase II trial using acalabrutinib for cGVHD is now underway (NCT04198922).

### 7.2 Treatment of recurrence

#### 7.2.1 Preemptive treatment of recurrence

Relapse in patients undergoing HSCT is strongly predicted by MRD positivity, BM CD34+ donor cell chimerism <80%, and persistent positivity for gene mutation or chromosomal gain or loss [74-75]. When the aforementioned risk factors appeared, we opted for preemptive treatment. DLI is generally regarded as the most expected method to achieve long-term remission in elderly patients (81), and it can dramatically decrease their risk of relapse (3-year RR was 32.4%) while also improving their probability of survival (3-year DFS was 50.3%) (82).

In order to figure out the most efficient and secure dose of lenalidomide, a prospective phase II clinical study is currently being conducted. Its goal is to assess the safety and viability of adding lenalidomide to AZA and DLI as a first preemptive treatment for AML patients who have relapsed after transplantation (NCT02472691). Moreover, some researchers have claimed that DLI and HMA can be used for the preemptive treatment of relapse, and relevant clinical research is still being done (NCT03662087).

Moreover, it has been demonstrated that AML patients face relapse after HSCT due to the downregulation of MHC class II genes implicated in *in vivo* antigen presentation, which circumvents the GVL effect. There is proof that interferon-gamma can change an AML cell’s phenotype (83). In the research by Xiaodong Mo et al., it was proved that the use of recombinant human interferon (IFN)-2b subcutaneous injection for six cycles as a preemptive treatment for patients who tested positive for MRD could continue to clear MRD. The 6-year cumulative RR after treatment was 13%, and the 6-year DFS was 83.1% (84). On the basis of the patient’s disease phenotype and genetics, we can also monitor the patients for molecular information and administer targeted drugs for preemptive treatment (85).

#### 7.2.2 Treatment after hematologic recurrence

Salvage therapy, which includes intensive chemotherapy, low-dose chemotherapy, secondary transplantation, DLI, enrolment in clinical trials, and palliative care, can be provided to patients who suffer a hematological relapse after HSCT (86). Due to their extremely poor physical performance, elderly patients who have relapse after HSCT should not get intensive chemotherapy or a secondary transplant.

After relapse, low-dose chemotherapy, including HMA or the addition of venetoclax, can be maintained in older patients with AML who had previously received treatment with it. Venetoclax with low-dose chemotherapy has been shown in several studies to increase the remission rate in relapsed patients. In this cohort, 43% of relapsed patients achieved CR/CR with CRi, and the half-year OS was 74% (87).

With only a 25% 2-year OS and a 49% 2-year RR in patients who achieved complete response, one study evaluating DLI therapy in patients with relapse after HSCT at a median age of 49 years had very poor results (81). Moreover, a number of studies support the use of DLI-based combination therapy. Venetoclax along with DLI can generate a CR rate of about 59%, according to a study by Odelia Amit et al. This has a major effect on those who have an early relapse post-transplantation (88). Moreover, DLI can be coupled with a range of other drugs, although they are all still in clinical studies, such as guadecitabine (NCT02684162), ruxolitinib and decitabine (NCT04055844), ipilimumab (NCT03912064), flotetuzumab (NCT04582864), siremadlin (NCT05447663), IFN- (NCT04628338), *etc.*

Several researchers have proposed that leukemia-specific T cells (mLSTs) specifically recognize multiple antigens expressed by AML cells, such as PRAME and WT1, compared with DLI. GVHD is minimized to low levels by mLSTs, which even have little to no effect in killing receptor normal cells. In total, 25 post-relapse patients were given an infusion of mLSTs by researchers; all of the patients experienced GVL effects, were well tolerated, and did not develop severe GVHD. Hence, mLSTs are both safer and more effective than DLI. When taken in conjunction with other medications, mLSTs increase the likelihood of long-term remission (89).

Because of the difficulty in finding the structural regions that CAR-T cells target in AML primary cells, cellular therapies like CAR-T cells have not been developed and used widely in AML. Clinical trials using CAR-T to treat patients who have relapsed after transplantation are still researched nevertheless (NCT04796441). Some relapsed patients may choose to take part in appropriate clinical trials or receive palliative care if they are not candidates for these treatments.

### 7.3 Management of other complications after HSCT

#### 7.3.1 Viral infection

Within 100 days of transplantation, the CI for viral infection was 70% (90). CMV is the most typical post-transplant viral infection. Patients who received MAC had a higher risk of getting infected with CMV than those who received NMA or RIC (91), and those who used much of cortisol hormone, cyclosporin, ATG, or other immunosuppression had a higher risk as well. Because their immune systems are more weakened and CMV disease has a worse prognosis, older people are more prone to get infect with it. Therefore, CMV prevention should be the main priority. The FDA approved letermovir in 2017 for prevention in post-transplant patients with serum CMV positivity. Recently developed CMV prevention drugs, such as maribavir and brindofovir, are currently undergoing drug trials. In addition, the body can directly rebuild CMV-specific cellular immunity after accepting an injection of virus-specific T cells, but this method is yet in the experimental stage (92) (NCT02985775). An ongoing clinical trial uses HSCT to immunize individuals against CMV in order to prevent an infection (NCT01588015). Relevant research show that younger patients have lower CIR when CMV reactivates early after HSCT (93, 94). Clinically, EBV reactivation post-HSCT also happens commonly. The research by Xiaojun Huang et al. (95) verified that worse 1-year OS and LFS were related to simultaneous CMV and EBV activation after transplantation, but none of the aforementioned conclusions was independently verified in elderly patients.

Human herpesvirus (HHV-6) infection is also common in post-transplant patients as well as to CMV. HHV-6 encephalitis is an important factor to morbidity and mortality after allo-HSCT. Patients receiving mismatched or unrelated donors, having low HHV-6 IgG titers prior to transplantation, having concurrent aGVHD, having previously been infected with EBV, and having poor T cell function were more likely to develop encephalitis at elevated levels of HHV6 deoxyribonucleic acid (DNA) in peripheral blood (96, 97). Patients with HHV-6B encephalitis had an OS rate of 58.3% compared with individuals without encephalitis who had an OS rate of 80.5% (98). Patients who are elderly have a worse prognosis. The incidence of HHV-6B encephalitis was not decreased by genicilovir and phosphoformic acid, according to prospective studies, and there are no effective drugs to prevent HHV-6 encephalitis (99, 100). To get serum HHV-6 DNA to turn negative, it is recommended to apply 90 mg/kg b.i.d. of intravenous foscarnet or 5 mg/kg b.i.d. of ganciclovir for at least 3 weeks (101, 102).

#### 7.3.2 Fungal infection and invasive fungal disease

After HSCT, the incidence of invasive fungal infection (IFI) can still range from 5% to 8% (103). Compared with younger patients, the incidence of invasive fungal disease (IFD) is significantly higher in this cohort of older individuals. The most frequent IFD was invasive aspergillosis (43%), which was followed by invasive candidiasis (28%) and zygomycosis (8%) (104).

Triazoles, which include posaconazole and isavuconzaole, are currently used very often for primary prophylaxis in patients who undergo HSCT (105). Echinocandins like caspofungin and micafungin are among the other first-line preventative medications. Sargramostim has been recommended by certain researchers as an additional regimen to avoid IFD for some individuals who are resistant to standard treatments. Sargramostim increases antifungal immune resistance in patients taking systemic corticosteroids and decreases the incidence and duration of neutropenia in cancer patients (106, 107).

Even though these medications are used to prevent IFD, roughly 5% of patients still get breakthrough IFD (108). These individuals frequently have severe comorbidities—relapsed or refractory hematologic malignancies are more prevalent in elderly patients—and have a high attributable mortality (109, 110). The early diagnosis of patients with breakthrough IFD is challenging; thus, as soon as these symptoms appear, a bronchoscopy should be done immediately, and bronoalveolar lavage samples should be taken for culture and galactomannan (GM) measurements. Moreover, lung computed tomography (CT) and serum GM can be utilized as supplemental diagnostic techniques for IFD (111, 112).

Voriconazole is recommended by the Infectious Diseases Society of America as the first-line treatment for IFD (113). According to one study, isavuconazole considerably decreased the incidence of hepatobiliary, ophthalmic, and cutaneous adverse effects in patients, but there was no difference in the overall success of treatment when compared with voriconazole (35% *vs*. 36%) (114). When compared with other triazoles, isavuconazole benefits from broad-spectrum efficacy, oral and injectable forms, predictable pharmacokinetics, and less adverse effects (115).

Most elderly patients have underlying conditions and comorbidities to varied degrees, which make them more susceptible to complications with the distribution, metabolism, absorption, and elimination of antifungals and other necessary medications as well as adverse drug responses (116). As a result, it is important to dynamically monitor the patient’s medicine concentration. The mean AUC 0–24 h amounted to 101 µg h/ml in patients with IFI. The minimal inhibitory concentration, according to the European Council for Antimicrobial Susceptibility Testing, is 1 g/ml or less (117). Those with mild to moderate hepatic insufficiency and renal insufficiency can continue utilizing standard doses, but recommendations for dosing in patients with Child–Pugh class C are lacking. Lastly, it is advised to reduce the dosage of isavuconazole and closely check the therapeutic agent while using isavuconazole with immunosuppressive drugs (118).

#### 7.3.3 Bacterial infection

After HSCT, elderly patients have weakened immune systems and are more vulnerable to bacterial infections. Antibiotics should be given as a preventative measure to patients who have bacterial infections that pose a high risk in clinical practice. For patients who have already appointed a bacterial infection, we should first administer an empirical antibiotic treatment, carry out pathogenic microorganism culture and drug susceptibility testing as soon as possible, and then modify the antibiotic treatment as the test results are conveyed to reduce the risk of organ injury brought on by hemodynamic disorders.

#### 7.3.4 Other late complications

Such late complications may affect vital organs like the heart, lungs, liver, and kidneys. Moreover, the incidence of secondary tumors climbed over time, reaching a 20-year CI of 21%, and its risk increased after HSCT, with a 5-year CI of 5% (119, 120). Around 10% of patients have cataracts (121). In addition to osteoporosis (122) and dyslipidemia (123), which affect more than half of elderly patients, avascular necrosis had a CI of 3.7% at 5 years and 5.4% at 10 years (124).

Cardiovascular complications after HSCT include ischemic heart disease, cardiomyopathy, vascular disease, *etc.* (125) The use of anthracycline drugs, cyclophosphamide-containing conditioning regimen, and chest radiotherapy can increase cardiotoxicity and thus increase the risk of cardiovascular complications after HSCT (126). Therefore, it is very important for elderly patients to select appropriate treatment programs based on cardiac function before and after HSCT and to follow up serum cardiac markers and echocardiography regularly after HSCT.

Another less common but life-threatening complication is cytokine release syndrome (CRS), which is present throughout the course of aGVHD. Severe CRS is more common in elderly patients, with an incidence of about 15% and a mortality rate of up to 24% (127). Patients with suspected CRS symptoms should undergo a comprehensive examination immediately. More importantly, the etiology of CRS should be distinguished (*i*.*e*., iatrogenic etiology, severe infection, *etc.*) (128) in order to select the appropriate treatment regimen (129). Some researchers have confirmed that an early combined blockade of interleukin 6 and human tumor necrosis factor γ is more effective than a single blockade therapy and can also preserve GVL effects (130). The treatment effect of tocilizumab monotherapy is also very effective. Some researchers believe that glucocorticoids may inhibit GVL effects, thus making it a second-line treatment (131).

The late mortality of transplant survivors along with their quality of life might be greatly affected by late complications. Subsequently, it is crucial to provide elderly patients undergoing HSCT with long-term follow-up, regular reviews, publicity, and education on the primary disease and its complications (132).

## 8 Conclusion

(1) Elderly patients with ECOG or HCT-CI ≥3 or with unfavorable cytogenetics should undergo allo-HSCT with caution.
(2) We recommend the use of RIC in elderly allo-HSCT patients. In terms of long-term survival, FM100 had an edge over FB, which had a lower NRM in the initial post-transplantation period.
(3) There are three main ways to prevent GVHD: PTCy, ATG, and *ex vivo* TCD. Combining these approaches with sirolimus, ruxolitinib, calcineurin inhibitors, MMF, *etc.*, may be more effective.
(4) Immunosuppressive drugs should be stopped quickly after transplantation in elderly patients. After HSCT, we should concentrate on regular disease monitoring in senior patients, and preemptive treatment of relapse is most critical. There are few trials on treatment after hematologic relapse, and it is ineffective.
(5) The most frequent viral infections after transplantation are CMV, EBV, and HHV6. The most crucial element for patients with breakthrough IFD is early diagnosis. The most crucial measures in the event of bacterial infections in elderly patients are early pathogenic examination and antibiotic sensitivity testing. In order to improve their QOL, elderly patients require long-term follow-up and routine reviews because they are more likely to experience late complications.

## 9 Outlook

Future research should concentrate on slashing RIC regimens that have excellent efficacy and safety, like clofarabine and FLAMSA, which are currently undergoing clinical trials, and consider combining these regimens to maximize their advantages. We can think about adjusting the schedule and dosage of traditional PTCy and ATG for GVHD prevention. Preemptive therapy has been a hot topic for post-transplant relapse, and in the future, more markers can be found to pinpoint when to begin preemptive therapy, which can be used to extend more preemptive treatment options like INF-γ. In order to determine whether treatments that are successful in younger patients can be used in elderly patients, more comprehensive randomized controlled trials in elderly AML patients are required in the future.

## Author contributions

SJ, RW, HC, and AZ writing—original draft preparation. HY, XL, WS, and LX writing—review and editing. All authors contributed to the article and approved the submitted version.
